# Proportion congruency effects: instructions may be enough

**DOI:** 10.3389/fpsyg.2014.01108

**Published:** 2014-10-06

**Authors:** Olga Entel, Joseph Tzelgov, Yoella Bereby-Meyer

**Affiliations:** ^1^Department of Psychology and Zlotowski Center for Neuroscience, Ben-Gurion University of the NegevBeer Sheva, Israel; ^2^Department of Brain and Cognitive Sciences, Ben-Gurion University of the NegevBeer Sheva, Israel

**Keywords:** Stroop, proportion congruent, item-specific congruency, conflict adaptation, control, learning

## Abstract

Learning takes time, namely, one needs to be exposed to contingency relations between stimulus dimensions in order to learn, whereas intentional control can be recruited through task demands. Therefore showing that control can be recruited as a function of experimental instructions alone, that is, adapting the processing according to the instructions before the exposure to the task, can be taken as evidence for existence of control recruitment in the absence of learning. This was done by manipulating the information given at the outset of the experiment. In the first experiment, we manipulated list-level congruency proportion. Half of the participants were informed that most of the stimuli would be congruent, whereas the other half were informed that most of the stimuli would be incongruent. This held true for the stimuli in the second part of each experiment. In the first part, however, the proportion of the two stimulus types was equal. A proportion congruent (PC) effect was found in both parts of the experiment, but it was larger in the second part. In our second experiment, we manipulated the proportion of the stimuli within participants by applying an item-specific design. This was done by presenting some color words most often in their congruent color, and other color words in incongruent colors. Participants were informed about the exact word-color pairings in advance. Similar to Experiment 1, this held true only for the second experimental part. In contrast to our first experiment, informing participants in advance did not result in an item-specific proportion effect, which was observed only in the second part. Thus our results support the hypothesis that instructions may be enough to trigger list-level control, yet learning does contribute to the PC effect under such conditions. The item-level proportion effect is apparently caused by learning or at least it is moderated by it.

## INTRODUCTION

The Stroop paradigm (Stroop, 1935; MacLeod, 1991) has been extensively used to investigate control of attention. In this paradigm, participants are asked to name the color of the ink of a color word and ignore the meaning of the stimulus word. Usually, participants respond slower when the word and the ink color are incongruent (e.g., GREEN written in red) compared to when the word is congruent with the ink color (e.g., RED written in red). This effect is known as *the Stroop effect* and it demonstrates effects of prepotent word reading processes on color naming performance (Cohen et al., 1990; MacLeod, 1991; Tzelgov et al., 1992).

*The proportion congruent (PC) effect*—an increase of the Stroop effect when the proportion of congruent stimuli increases—is frequently taken as a marker of conflict adaptation in the Stroop task (i.e., participants are able to adapt to conflict encountered in the task by adjusting attention away from the source of conflict; Logan and Zbrodoff, 1979; Logan, 1980; Lowe and Mitterer, 1982; Cheesman and Merikle, 1986; Lindsay and Jacoby, 1994).

Botvinick et al. (2001) modeled the control of the Stroop effect by extending Cohen et al.’s (1990) work, thereby providing a possible explanation for the PC effect^1^. According to the conflict monitoring model of Botvinick et al. (2001), control is triggered by a module responsible for detecting conflicts in information processing; namely, the conflict monitoring unit (assumed to be located at the ACC). This unit calculates the amount of conflict at the response layer and accordingly increases the input from the relevant task demand (color naming) units when the level of conflict is high. This mechanism measures the level of conflict on each trial and then the cognitive system uses this conflict information to adjust attention (i.e., *conflict adaptation*).

In the Stroop task, incongruent items generate more conflict than congruent items. Namely, higher proportions of incongruent items give rise to higher levels of conflict, which in turn result in increased cognitive control via the activation of the relevant task demands (color naming), leading to a decreased Stroop effect.

However, recent findings have challenged this widely accepted model and the conflict adaptation theory overall. This challenge started with the finding that the PC effect can be item-specific. The *item-specific proportion congruent (ISPC) effect*—larger congruency effect for color words presented mostly congruently than for those presented mostly incongruently (Jacoby et al., 2003; Schmidt et al., 2007)—pointed to possible involvement of learning processes in the proportion effect. Jacoby et al. (2003) introduced the ISPC task by manipulating PC between items instead of between participants or between blocks. That is, some words (e.g., BLUE and RED) were presented most often in their congruent color (high PC), while others (e.g., GREEN and YELLOW) were presented most often in an incongruent color (low PC). A larger congruency effect was observed for high, relative to low, PC items. The conflict adaptation account suggests that PC effects are due to modulation of attention to the word as a reaction to the general conflict level in the task as a whole, but given that high and low PC trials are intermixed in the item-specific task, one cannot know in advance whether one needs to attend or not attend to the word. Therefore, it cannot explain item-specific effects. Learning accounts conversely propose that the cognitive system learns how to respond to a specific condition, thus explaining item-specific proportion effects. Therefore, conflict adaptation does not have to be assumed.

Given this problem, Blais et al. (2007; see also Verguts and Notebaert, 2008, 2009; Blais and Verguts, 2012) proposed that learning processes may contribute to conflict adaptation. In particular, Blais et al. (2007) modified Botvinick et al.’s (2001) model by allowing the modulation to be condition-specific, that is, by modulating the connections between a specific condition [e.g., the stimulus color (Bugg et al., 2008) or location^2^ (Crump et al., 2006)] and the required response according to their co-occurrence. Such a model allows for simulating ISPC effects. Verguts and Notebaert (2008, 2009) noted that the conflict monitoring model (Botvinick et al., 2001) and the Blais et al. (2007) version of it clearly specify when extra control should be exerted, but not where. In particular, response conflict warns the cognitive system that it should be attentive, thus specifying when it should be activated. The conflict monitoring model further postulates that this is done by increasing activation of the currently relevant task representation (task demand unit). However, how the control system knows which stimuli are more conflicting than others [e.g., how the systems knows that red and blue are mostly congruent (MC), while yellow and green are mostly incongruent (MI), i.e., where to intervene] is left unspecified. As a solution to this problem, Verguts and Notebaert (2009) proposed a model in which Hebbian learning provides the mechanism for binding specific stimulus–response combinations, suggesting that the modulation of cognitive control might be the result of interactions between arousal and online learning processes.

While flexible conflict adaptation models (Blais et al., 2007; Verguts and Notebaert, 2009) as presented above can provide an explanation for ISPC effects by showing that control adaptation can be applied under specific conditions, such effects can be also explained by a pure stimulus–response (S–R) contingency-learning mechanism (Schmidt et al., 2007, 2010; Schmidt and Besner, 2008; see also Mordkoff, 1996). According to the contingency-learning account, participants learn the associations between certain words and certain responses and thus frequently appearing conditions are responded to much quicker. In tests of this account, as in an ISPC experiment, one set of words is presented mostly in their congruent color (e.g., GREEN written in green), and another set is presented mostly in their incongruent color (e.g., BLUE written in yellow), and thus participants are able to associate certain words with certain responses (e.g., the word green with the response green, the word blue with the response yellow).

Schmidt and Besner (2008) claimed that the standard PC experiments confound item-specific and list-level^3^ PC effects. Because all stimuli are presented most often in their congruent color in the MC condition, and most often in their incongruent color in the MI condition, these contingency biases are capable of producing a PC effect on their own. Bugg and Chanani (2011; see also Bugg et al., 2011), however, proposed that both contingency learning and conflict adaptation may contribute to the proportion effect in the Stroop task. They demonstrated that list-wide proportion effects cannot be fully explained by item-specific mechanisms (cf. Hutchison, 2011). Bugg and Chanani suggested that participants may not implement list-wide control when associative learning provides a reliable and efficient means for responding (e.g., Bugg et al., 2008; Blais and Bunge, 2010). In their word-picture Stroop experiment, they increased the set of possible responses on incongruent trials, that is, they generated more contingent response options, which made associative learning less effective. The researchers found a list-level proportion congruency effect for 50% congruent items, showing that list-level proportion congruency effects could be observed independently of ISPC effects. Bugg (2014) demonstrated similar results in a color-word Stroop task. In her study, participants showed no evidence of increased control in high relative to low conflict context when they were able to rely on item-specific S–R associations to respond to the majority of trials (Experiments 1B and 2A). By contrast, when this was not a reliable approach, due to there being multiple possible responses on incongruent trials (i.e., a four-item biased set), increased use of control was observed in the high relative to the low conflict context (PC effect in Experiments 1A and 2B).

Recently, Schmidt (2013a) proposed a temporal learning-based explanation of list-wide PC effects. According to his proposal, participants may learn when to respond in specific conditions rather than what to respond (i.e., contingency learning). Such *temporal learning* results in biasing response retrieval times in specific conditions. Namely, in the high PC conditions, congruent trials are responded to faster, so the high frequency of quick responses leads participants into a rapid pace of responding to congruent trials, with a penalty to the infrequent incongruent trials. In contrast, in the low PC conditions, most previous responses are slow, leading to a slower expectancy. Incongruent trials benefit from the slower expectancy, thus leading to a smaller Stroop effect.

## THE PRESENT STUDY

The associative learning accounts that propose learning-based modulation of conflict adaptation (i.e., Blais et al., 2007; Verguts and Notebaert, 2008, 2009; Blais and Verguts, 2012), as well as those proposing that (contingency) learning alone can account for the PC and the ISPC effects (Schmidt et al., 2007, 2010; Schmidt and Besner, 2008; Schmidt, 2013b), suggest that gaining experience with the stimuli (S–R) relations is crucial in order to learn. As described earlier, the ISPC effect was examined in order to reveal the contribution of learning. We examined if gaining experience is a necessary condition for the PC effect. The conflict monitoring model (Botvinick et al., 2001), as well as the computational model of Blais et al. (2007), does not specify where extra control has to be exerted. It does suggest, however, that once the system knows there is a conflict, it recruits control. This allows us to assume that “knowing” may be enough and the actual exposure to the task (i.e., experiencing the S–R relations) is not always necessary to generate control. Therefore, showing that PC effects can be observed as a function of experimental instructions alone, when participants receive no practice, is evidence of the existence for control recruitment in the absence of learning.

The idea that instructions alone are not enough in order to learn is supported by Schmidt and De Houwer’s (2012) findings. These researchers aimed to reveal whether contingency awareness resulting from instructions can aid performance in an implicit learning task, such as the color-word contingency learning task. In their second experiment, three color-unrelated words were presented most often in a particular color (e.g., “plate” most often in green, “month” most often in red, and “under” in yellow). In addition, they manipulated the experimental instructions. Half of the participants were told the word-color contingencies in advance and half were not. The researchers showed that when the contingency instructions were given, but no contingencies were actually present, no contingency effect was found. By contrast, Meiran et al. (2012) proposed recently that application of novel plans that have never been executed before is not only possible but may actually represent the typical scenario of control adaptation. Similarly, Verbruggen et al. (2014) proposed that participants are able to derive action rules from instructions and immediately perform a task that they have never done before as a prepared or intention- based reflex.

Based on these findings, the aim of the present study was to distinguish between the effects of control recruitment by instructions (henceforth *control by instructions*) and those of learning.

## EXPERIMENT 1

We aimed to differentiate between control by instructions and learning by manipulating the information given at the outset of the experiment. Half of the participants were informed that most of the stimuli would be congruent, whereas the other half were informed that most of the stimuli would be incongruent. This held true for the stimuli in the second part of the experiment, however, in the first part the proportion of the two stimulus types was equal; therefore it was impossible to learn the proportions during the first part. If control by instructions does exist, we would expect to find a significant PC effect from the very beginning of the experiment. If the PC effect also reflects learning, the effect should be larger in the second part of the experiment. Finally, if PC is caused exclusively by learning, it should appear only in the second half of the experiment.

### METHOD

#### Participants

Twenty-eight students at Ben-Gurion University of the Negev, who were native speakers of Hebrew, participated in the experiment. All had normal or corrected-to-normal eyesight. Participation in the experiment was in partial fulfillment of course requirements. All participants gave written informed consent. The experiment was approved by the ethics committee of the Psychology Department at Ben-Gurion University of the Negev.

#### Stimuli

We used four colors in the experiment: red, green, blue, and yellow. The name of each of these colors in Hebrew consists of four letters. We generated the congruent stimuli by printing each of the four color names in its own color. We generated the incongruent stimuli by printing each color name in ink colors of the three other colors. The stimuli were presented on a 17′′ widescreen CRT monitor with a resolution 1024 × 768, in bold-faced 18-point Courier New font. Data collection and stimuli presentation were controlled by E-Prime software (Psychology Software Tools, Pittsburgh, PA, USA) on a Dell computer with an Intel Pentium 4 central processor. The two types of stimuli (congruent or incongruent) were randomly ordered.

#### Design and procedure

We created two experimental conditions. Half of the participants were informed that most of the stimuli would be congruent, whereas the other half were told that most of the stimuli would be incongruent. This held true for the stimuli in the second part of the experiment [congruent to incongruent ratio (C/I) = 80/20 or 20/80 in accordance with the instructions given] but in the first part the proportion of the two stimulus types was equal; therefore it was impossible to learn the proportions during the first part (C/I ratio = 50/50). Fourteen participants were randomly assigned to each of the experimental conditions. Participants were tested individually. At the beginning of the experiment, the task was explained to the participants, who were asked to ignore the written word and name the ink color as fast as possible without making errors. Depending on the experimental group they were allocated to, participants were informed what the distribution of the stimuli to be presented would be: (English translation) *In this experiment, you will see congruent (the word and the ink color are congruent, e.g., RED printed in red) and incongruent (the word and the ink color are incongruent) stimuli. Note that most of the stimuli will appear as congruent (/incongruent) stimuli.*

There were no practice trials and no breaks between the two experimental parts. Each experimental part consisted of 120 trials. The participants sat opposite to the display screen. The stimuli were presented in the center of the screen, at ∼80 cm from the participant’s eyes. Each trial began with a fixation point presented for 500 ms (a white plus sign at the center of a black screen). After that, the stimulus appeared and remained in view until the participant’s response into a microphone, which stopped the timer and removed the stimulus from the screen. Reaction time (RT) in milliseconds was measured by the computer from the stimulus onset until the participant’s response. A keypress by the experimenter initiated the next trial. Responses were scored as errors if the initial consonant sound indicated a color other than that of the current trial. The experimenter typed in the vocal response of the participant on one of four keys so that the computer could evaluate errors.

The instructions given to participants (“most of the stimuli would be congruent/ incongruent”) were manipulated between participants. Stimulus type (congruent or incongruent) and the part of the experiment (first or second) were manipulated within participants (see **Table 1** for details). RT was the main dependent variable in the experiment.

**Table 1:** Experimental design.

Experimental group	Instructions	Part	Congruent to incongruent ratio	Number of trials	Total
1	Most stimuli	1	50/50	120	240
	congruent	2	80/20	120	
2	Most stimuli	1	50/50	120	240
	incongruent	2	20/80	120	

### RESULTS AND DISCUSSION

For each participant, mean RTs of correct responses and of the percentage of errors (PEs) in each experimental condition were calculated. RTs of error trials were omitted (less than 2% of all responses) as were RTs slower than 2,500 ms and faster than 250 ms. All effects were tested at a significance level (α) of 0.05.

**FIGURE 1:**
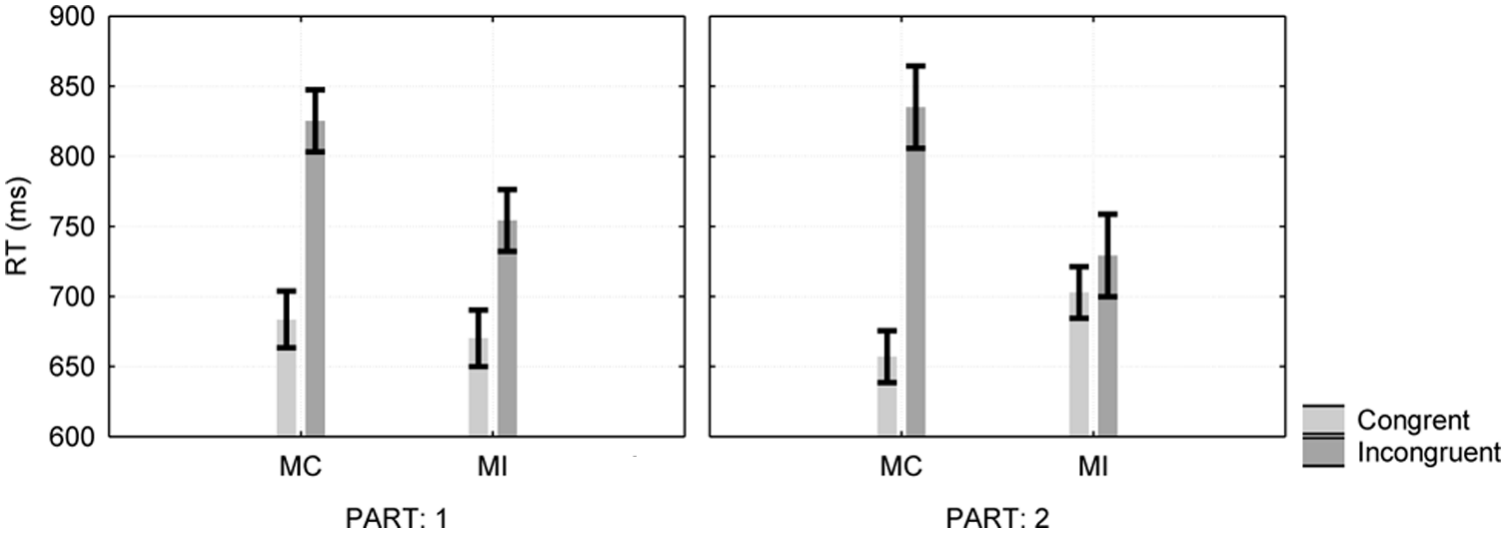
**Proportion congruent effect as a function of instruction type and experimental part in Experiment 1.** Error bars are one standard error of the mean. MC, mostly congruent; MI, mostly incongruent.

A three-way ANOVA (analysis of variance) mixed-factor model with stimulus type and part of the experiment as within-participant factors, and type of instructions as a between-participant factor, revealed a significant main effect for stimulus type, *F*(1,26) = 104.2, MSE = 3,107, $\eta_{p}^{2}$ = 0.8. The two-way interaction between stimulus type and instructions was significant, *F*(1,26) = 24.65, MSE = 3,107, $\eta_{p}^{2}$ = 0.49, as was the three-way interaction between the stimulus type, part and the instructions, *F*(1,26) = 12.36, MSE = 1,250, $\eta_{p}^{2}$ = 0.32 (see **Figure 1**).

Further analysis revealed significant simple interactions between instructions and stimulus type, both in part 1 where the proportions of the color words were equal, *F*(1,26) = 11.16, MSE = 1,039.87, $\eta_{p}^{2}$ = 0.3, and in part 2 where we changed the proportions in accordance with the instructions, *F*(1,26) = 24.24, MSE = 3,317.04, $\eta_{p}^{2}$ = 0.48, revealing smaller Stroop effects in the C/I = 20/80 conditions (142 ms for MC vs. 84 ms for MI in part 1, and 178 ms for MC vs. 26 ms for MI conditions in part 2).

In the current design, the part of the experiment and the proportions that the participants were exposed to were confounded; the first and second experimental parts differed not only in proportions but also in length of exposure to experimental stimuli and in the amount of fatigue experienced by the participant. To test the hypothesis that the time location in the experiment *per se* influenced behavior, were analyzed the first part of the experiment after adding location within the first part (i.e., first vs. second half) to the design. Splitting the first part did not moderate the effects of congruency, instructions or their interactions (*F* < 1). This finding supports the claim that the increase in the PC effect (by 94 ms) in the second part of the experiment is due to learning. As one can see in **Figure 1**, responses for congruent stimuli were 46 ms faster when most of the stimuli were congruent, *F*(1,26) = 13.3, MSE = 922.34, $\eta_{p}^{2}$ = 0.34, and incongruent stimuli were 106 ms faster when most of the stimuli appeared as incongruent, *F*(1,26) = 6.47, MSE = 12,102, $\eta_{p}^{2}$ = 0.20.

The error rate was very low, averaging 1.24%. A three-way repeated measures ANOVA with stimulus type and part as within-participant factors, and type of instructions as a between-participant factor, revealed a single significant main effect for stimulus type, *F*(1,26) = 8.45, MSE = 2.97, $\eta_{p}^{2}$ = 0.24, showing more errors for the incongruent stimuli.

These results, as those proposed by Bugg (2014; see also Bugg and Chanani, 2011), suggest that both learning and control adaptation via mere instructions may contribute to the proportion effect in the Stroop task. Namely, our results show that in addition to stimulus–response associative learning, control contributes to the proportion effect.

## EXPERIMENT 2

Experiment 1 demonstrated that pro-active control can be activated by instructions in the absence of learning. In the first experiment we manipulated the list-level proportions (i.e., most of the stimuli appeared as congruent or incongruent trials), but we did not change the item-specific proportions. Therefore, the purpose of our second experiment was to discover whether the ISPC effect could be observed as a function of experimental instructions alone, thus indicating item-level control recruitment.

We manipulated the item-specific proportions, generating two color sets–color words presented mostly in their congruent color, and color words presented mostly in their incongruent color. Participants were informed about the exact word-color contingencies in advance. Similar to Experiment 1, this held true only for the second part of the experiment, while in the first experimental part the item-specific proportion was equal. Therefore it was impossible to learn the proportions during the first part. The list proportion congruency was held constant. If item-level control by instructions does exist, we would expect to find a significant ISPC effect from the very beginning of the experiment. If the ISPC effect also reflects learning, the effect should be larger in the second part of the experiment. Finally, if the ISPC effect is caused exclusively by learning, it should appear only in the second half of the experiment.

### METHOD

#### Participants

Thirty-four students at Ben-Gurion University of the Negev, who were native speakers of Hebrew, participated in the experiment. All had normal or corrected-to-normal eyesight and had not participated in Experiment 1. Participation in the experiment was in partial fulfillment of course requirements. All participants gave written informed consent. The experiment was approved by the ethics committee of the Psychology Department at Ben-Gurion University of the Negev.

#### Stimuli and procedure

We used two sets of color words (i.e., red and blue vs. yellow and green). In the first part of the experiment, each color word was presented 30 times and the item-specific proportion was equal for the two sets (e.g., red and blue were presented half of the time in their congruent color (i.e., in 15 trials each) and the other half in their incongruent color). In the second part, we varied the item-specific proportions: for the first set, each color word was presented in its congruent color in 24 trials (80%) and in the other color from that set in 6 trials (20%) to produce the MC condition. For the second set, these rates were reversed to produce the MI condition (i.e., each color word was presented as an incongruent stimulus in 24 trials and as a congruent stimulus in 6 trials). Thus, overall, in the experiment there were 120 congruent trials and 120 incongruent trials (60 congruent and 60 incongruent stimuli in each block), with each color and color-word appearing equally often, while the item-specific proportion was changed only in the second experimental part. Assignment of color sets to the MC and MI conditions was counterbalanced across participants.

At the beginning of the experiment, the task was explained to the participants, who were asked to ignore the written word and name the ink color as fast as possible without making errors. In addition, participants were informed what the distribution of the stimuli to be presented would be. As in Experiment 1, they received instructions telling them of the word-color contingencies involved in the task. One group was instructed that red and blue would appear mostly as congruent stimuli, while yellow and green would appear mostly as incongruent stimuli: (English translation) *In this experiment, you will see color words printed in colors. Note that RED and BLUE will appear mostly as congruent stimuli (the word and the ink color are congruent), while YELLOW and GREEN will appear mostly as incongruent (the word and the ink color are incongruent) stimuli*. The other group was instructed exactly the opposite, that is, that yellow and green would appear mostly as congruent stimuli while red and blue would appear mostly as incongruent stimuli. There were no practice trials and no break between the two experimental parts. Each experimental part consisted of 120 trials.

Three independent variables—condition (MC or MI), stimulus type (congruent or incongruent), and experimental part (1 or 2)—were manipulated between participants (see **Table 1** for details). RT was the main dependent variable in the experiment.

### RESULTS AND DISCUSSION

For each participant, mean RTs of correct responses and of the PE in each experimental condition were calculated. RTs of error trials were omitted (less than 1% of all responses) as were RTs slower than 2,500 ms and faster than 250 ms. All effects were tested at a significance level (α) of 0.05.

**FIGURE 2:**
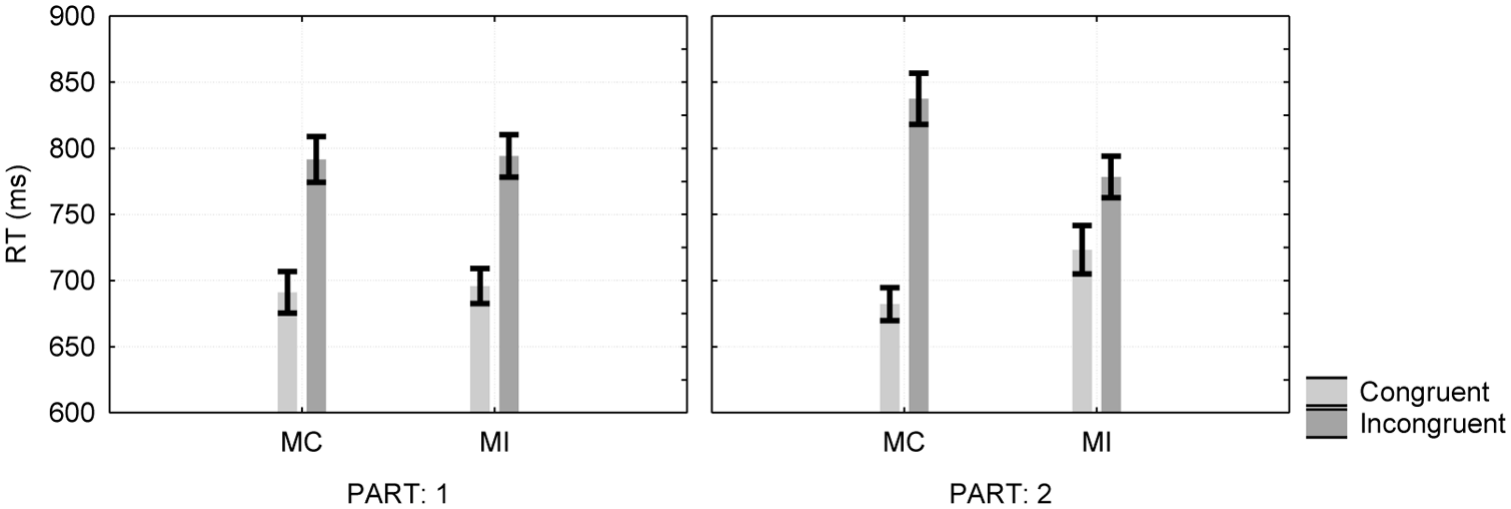
**Item-specific proportion congruent effect as a function of instruction type and experimental part in Experiment 2.** Error bars are one standard error of the mean. MC, mostly congruent; MI, mostly incongruent.

A three-way repeated measures ANOVA with condition, stimulus type, and part as within-participant factors revealed a significant main effect for stimulus type, *F*(1,33) = 151.2, MSE = 4,707, $\eta_{p}^{2}$ = 0.82. The two-way interaction between condition and stimulus type was significant, *F*(1,33) = 29.53, MSE = 1,516, $\eta_{p}^{2}$ = 0.47, as was the three-way interaction between the condition, stimulus type and part, *F*(1,33) = 28.9, MSE = 1,412, $\eta_{p}^{2}$ = 0.47 (see **Figure 2**).

The first part of the experiment revealed no difference between the congruency effects in the two proportion conditions, *F* < 1. This finding implies that informing the participants about the word-color contingencies without giving them the opportunity to learn the S–R relations was not enough to produce the ISPC effect. In order to reassure this additive pattern, we also computed the Bayesian posterior probabilities (see Wagenmakers, 2007; Campbell and Thompson, 2012). We estimated the posterior probabilities of *p* (H_0_ | D; i.e., the posterior probability of null effect of instructions) and of *p* (H_1_ | D; i.e., the posterior probability that instructions were enough to modulate control) as 0.85 and 0.15 for H_0_ and H_1_ respectively, leading to dBIC of 3.5, which according to Campbell and Thompson (2012), is substantial evidence for H_0_. Thus, it is apparently not enough to provide information that processing the meaning of stimuli in specific colors is harmful to performance.

In contrast to the first part of the experiment, we observed a significant simple interaction between condition and stimulus type in the second part, where we changed the specific item proportions in accordance with the instructions, revealing a significant ISPC effect, *F*(1,33) = 38.14, MSE = 2,242.52, $\eta_{p}^{2}$ = 0.54. Further analysis revealed faster responses (by 41 ms( for the congruent stimuli in the MC condition, *F*(1,33) = 9.04, MSE = 3,178.98, $\eta_{p}^{2}$ = 0.22, and faster responses (by 59 ms) for the incongruent stimuli in the MI condition, *F*(1,33) = 36.04, MSE = 1,653.08, $\eta_{p}^{2}$ = 0.52.

The error rate was very low, averaging 1%. A three-way repeated measures ANOVA with item type, stimulus type, and part as within-participant factors revealed faster responses for congruent stimuli, *F*(1,33) = 33.65, MSE = 4.24, $\eta_{p}^{2}$ = 0.5. The two-way interaction between item type and stimulus type was significant, *F*(1,33) = 6.4, MSE = 3.96, $\eta_{p}^{2}$ = 0.16, revealing more errors for incongruent stimuli in the MC condition rather than in the MI condition, *F*(1,33) = 33.65, MSE = 4.73, $\eta_{p}^{2}$ = 0.13. Less errors were also observed for congruent trials in the MC condition than in the MI condition, however, the difference was not significant, *F*(1,33) = 1.64, MSE = 2.6, $\eta_{p}^{2}$ = 0.05.

Observing a significant ISPC effect when item-specific proportions are varied, resulting in color-word contingency, implies that this effect reflects associative learning. Therefore our results support the notion that learning processes are important (e.g., Schmidt and Besner, 2008; Verguts and Notebaert, 2008, 2009), showing that in contrast to the PC effect, the ISPC effect is not observed in the absence of learning, that is, experiencing S–R contingencies is crucial in order to be able to adapt performance.

## GENERAL DISCUSSION

In our first experiment, we informed half of the participants that most of the stimuli would be congruent, whereas the other half were told that most of the stimuli would be incongruent. This held true for the stimuli in the second part of the experiment, but in the first part the proportion of the two stimulus types was equal. A significant PC effect was found in both parts of the experiment, but it was larger in the second experimental part. In our second experiment, we manipulated the item-specific proportions, while the list-level proportion was held constant. Participants were told word-color contingencies in advance but the information, similar to in Experiment 1, was valid only for the second experimental part. In contrast to the list-wide PC effect in Experiment 1, in Experiment 2 the ISPC effect was found only in the second experimental part, where we varied the item-specific proportions.

Our results showed that instructions may be enough to trigger list-level control, thus supporting Cohen-Kdoshay and Meiran (2009) who showed that instructions can be implemented with a high degree of accuracy even on the very first trial. This raises an important question: *How do instructions influence behavior?* Recently, Ramamoorthy and Verguts (2012; see also Doll et al., 2009) suggested a possible computational model of applying instructions. In their model they distinguish between instructions leading to rule-based “learning” and actual exposure to the task stimuli. According to the model, instructions are acquired [apparently by the prefrontal cortex (PFC)] before the actual exposure to the task. Upon repeated application, the basal ganglia (which learn more slowly but execute more quickly) pick up the appropriate stimulus–response mapping by Hebbian learning.

It is important to note that in contrast to our first experiment, in Experiment 2 the ISPC effect was found only in the second experimental part, showing that this effect cannot be produced solely by prior information about the word-color contingencies. We assume that a possible reason for this difference can lay in the difficulty of the instructions. Learning all the item-specific pairings in the ISPC task is much harder than learning “most of the stimuli will be presented in their congruent/incongruent color,” therefore, in our first experiment adapting performance via instructions was much easier than in our second experiment and also in Schmidt and De Houwer’s (2012) study. This explanation is line with Meiran et al.’s (2012) claim that automatic applications of novel (never executed before) plans are possible only if the task instructions are not too complicated.

To conclude, our results shed new light on the relations between control and associative learning, showing that both processes can take part in the modification of the Stroop phenomenon. We show that under specific (relatively simple) conditions, practice is not necessary for the emergence of the PC effect, from which it follows that control adaptation may lead to such effects. While the conflict monitoring model (Botvinick et al., 2001) and all its later versions (e.g., Blais et al., 2007) do not show how conflict adaptation can be activated by instructions alone, such models could be easily extended to include adaptation on the basis of instructions as shown by Cole et al. (2010) and Ramamoorthy and Verguts (2012). Furthermore, it could also be argued that the increase in the PC effect in the second part of Experiment 1 can also be explained by conflict adaptation, assuming that changes in the color-word contingencies change the actual experienced level of conflict, thereby increasing the PC effect. This, however, cannot explain the results of Experiment 2 without assuming a learning mechanism that directs attention to high conflict conditions (e.g., Hebbian learning as suggested by Verguts and Notebaert, 2008). Thus, it seems that several mechanisms are involved in the PC effect.

## Conflict of Interest Statement

The authors declare that the research was conducted in the absence of any commercial or financial relationships that could be construed as a potential conflict of interest.

## Footnotes

1 It should be clear that in the absence of neutral stimuli (as in the designs of Logan and Zbrodoff, 1979; Logan, 1980; Lowe and Mitterer, 1982; Lindsay and Jacoby, 1994) the PC effect means that the proportion of incongruent stimuli out of the total number of stimuli in a given block decreases as the proportion of congruent stimuli increases. Botvinick et al. (2001) modeled the control of the Stroop effect by simulating the results of Tzelgov et al. (1992). Note that these researchers employed neutrals in their design and their manipulation was based on changing the proportion of color word vs. neutrals while holding the ratio of congruent to incongruent stimuli constant. This implies that in this case conflict adaptation was led by the proportion of the conflicting (incongruent) stimuli out of the total number of stimuli in each experimental block.

2 Blais et al. (2007) did not simulate the context-specific proportion congruent (CSPC) effect, but we believe this could probably be done in their model.

3 The term list-level PC (in contrast to item-specific PC) refers to a situation in which the congruent to incongruent ratio is held constant for all color words included in a given block.
